# Seroprevalence of African swine fever in pigs for slaughter in Leyte, Philippines

**DOI:** 10.5455/javar.2024.k748

**Published:** 2024-03-28

**Authors:** Harvie P. Portugaliza

**Affiliations:** Department of Veterinary Clinical Sciences, College of Veterinary Medicine, Visayas State University, Visca, Baybay City, Philippines

**Keywords:** African swine fever, ELISA, epidemiology, prevalence

## Abstract

**Objective:**

This study aims to determine the seroprevalence of African swine fever (ASF) in pigs for slaughter in Leyte, Philippines. It underpins the concept that recovered and infected pigs from ASF are likely sent for slaughter to avoid perceived economic losses.

**Materials and Methods:**

A cross-sectional study was conducted from March to June 2023 in eight abattoirs, each representing both a city and a municipality in Leyte province. A total of 218 blood samples from 78 farms were examined for ASF virus (ASFV) (VP72) antibodies using a blocking enzyme-linked immunosorbent assay (ELISA) method. Descriptive and seroprevalence analyses were performed.

**Results:**

ASF antibodies were detected in pigs for slaughter from abattoirs in Baybay and Ormoc cities, showing a seroprevalence of 3.57% (1/28 pigs) and 2.27% (1/44 pigs), respectively. An apparent absence of ASF antibodies was observed among pigs for slaughter in Isabel, Villaba, Abuyog, Kananga, Dulag, and Macarthur. The farm-level seroprevalence was 2.56% (95% CI: 0.71%–8.88%), while the pig-level seroprevalence was 0.91% (95% CI: 0.25%–3.27%).

**Conclusion:**

Detecting ASF antibodies among pigs for slaughter implies exposure to the virus from the farm of origin. This means that, on some farms, ASF remains unreported or undiagnosed. Active surveillance is needed for early case detection and rapid response to control the spread of ASF in the country.

## Introduction

African swine fever (ASF) is a highly contagious disease in pigs, with mortality rates reaching 100% in lethal genotypes of the ASF virus (ASFV) [1]. ASFV is remarkably infectious owing to virion stability in the environment, pork meat, and processed products, spreading quickly and easily across susceptible pigs. An effective vaccine against ASFV has been elusive due to the complexity of the viral particles, the gap in knowledge on virulence genes, host immunity, host-pathogen interaction, and inefficient methods for viral culture and testing. Therefore, ASF intervention without a commercially available vaccine relies on effective preventive and control measures designed to tailor the specific geographical area [2,3].

The spread of the ASF virus has become a major concern across continents. In 2018, ASF was first reported in China and has since spread to various countries in Asia, including the Philippines [3,4]. In the Philippines, ASF has devastated 67 out of 81 provinces, causing at least 50 million pesos in losses in local pork production due to pig mortality since its first outbreak in 2019 [5]. With no signs of stopping from taking over the country’s pork industry, the Philippine President proclaimed a state of calamity on May 11, 2021, to augment government efforts in containing the spread of ASF and assisting local pig farmers (Presidential Proclamation No. 1143) [6]. The Philippine government has instituted several measures, including public awareness, border restrictions, and a culling-restocking strategy. However, previous work in many countries that followed these measures has not been successful due to the non-compliance of stakeholders and the lack of local understanding of ASF epidemiology [7,8].

In most ASF outbreaks, determining the start of infection is challenging due to the complexity of ASF transmission pathways. The non-reporting of outbreaks to proper authorities and the potential selling of pigs from outbreak zones have been shown to contribute to ASF spread [9]. This scenario is not uncommon in the Philippines, with the previous study documenting the potential non-reporting of outbreaks and the sale of potentially infected pigs [10]. With logistic challenges for regular ASF surveillance, testing pigs brought for slaughter is a viable option as an indicator of unreported outbreaks in areas affected by ASF. Therefore, this study aimed to determine the seroprevalence of ASF in pigs delivered for slaughter in municipal and city abattoirs in Leyte, Philippines, underpinning the idea that ASF-recovered and infected pigs sent for slaughter carry antibodies against the ASF virus and thus become potential spreaders of ASF in the region [11,12]. This is the first research-based study in the Visayas to determine the prevalence of ASF in apparently healthy pigs delivered to various abattoirs.

## Materials and Methods

### Ethical approval

This study was approved by the CVM-VSU Research Ethics Committee. All collected data were treated according to Philippine R.A. 10173, or the Data Privacy Act of 2012. Pig sample collections were guided by the principles of the Animal Welfare Act of the Philippines (RA 8485).

### Study site

Leyte is located in the Eastern Visayas Region of the Central Philippines. It is the largest and oldest province in the region, occupying around 27%, or 571,276 hectares, of the overall land area. The province is divided by a cordillera that separates the western and eastern lowlands. The peaks in Leyte range from 2,295 to 3,280 feet in elevation, extending from north to south. The province is divided into five congressional districts and has 1,503 barangays. It has 40 communities and two commercial cities, Ormoc and Baybay [13].

### Study and sampling design

**Figure 1.**
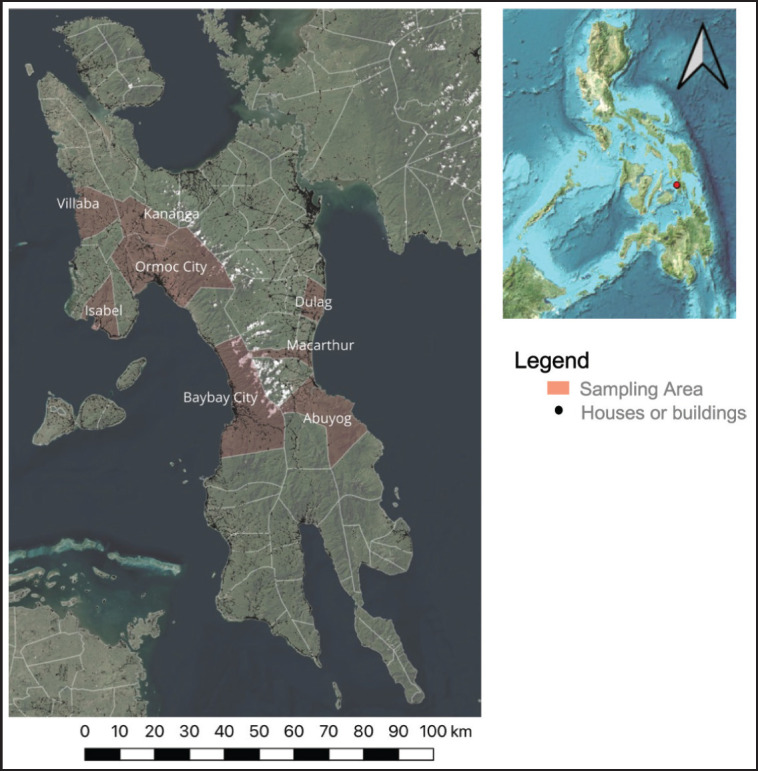
Study sites. Municipalities and cities in Leyte, Philippines, where blood samples were collected in abattoirs for ASF seroprevalence. The map was created using QGIS (qgis.org/en/site/)

A cross-sectional study was conducted to determine the seroprevalence of ASF among pigs for slaughter. A conventional sampling was performed in the local abattoirs of eight municipalities and cities from March to June 2023 (Fig. 1), comprising Villaba, Kananga, Ormoc City, Isabel, Baybay City, Abuyog, Macarthur, and Dulag. Of note, the sampling design was conventional (i.e., choosing only those with abattoirs) because most municipalities on Leyte Island have no registered abattoirs. Therefore, the inclusion criteria comprised municipalities and cities with operational abattoirs, pigs for slaughter present in the holding pen or undergoing the slaughtering process, and the voluntary participation of pig owners and abattoir management for sample collection. The number of samples ranged from 21 to 44 pigs, covering 1 to 19 pig farms (Table 1).

### Sample collection

Blood collection was carried out in two ways, depending on the procedures of each abattoir and the personnel available during sample collection. The first method is collecting blood samples from pigs in the holding pen. Pigs were restrained securely against the corner, with the head held high, exposing the neck to locate the jugular furrow where the jugular vein is situated. The blood collection site was disinfected with 70% ethanol. Using a 5 ml syringe (needle size 1.5 inches; 16 gauge), 3 ml of blood was collected and transferred to an appropriately labeled collection tube without the anticoagulant (red top blood collection tube) [14]. In the second method, blood was taken during pig slaughter (exsanguination stage). This involved restraining, stunning the pigs, incising the neck, and collecting the midstream blood into a 3 ml collection tube without an anticoagulant. Properly labeled samples were stored in a cold transport container for further processing in the College of Veterinary Medicine (CVM) Diagnostic Laboratory.

### Pig serum preparation and blocking ELISA method

Blood samples were allowed to sit for 30 min at room temperature, after which the sample was centrifuged for 10 min at 2,500 rpm [15]. Non-hemolyzed serum samples were transferred to a 2 ml microcentrifuge tube and stored at −80°C until used for ELISA. The ASFV antibodies against VP72 proteins were detected using a blocking ELISA method, following the manufacturer’s protocol (INGEZIM PPA COMPAC 11.PPA. K3; Ingenasa, Spain). VP72 is a major antigenic ASFV structural protein [16].

### Abattoir and pig data collected

The abattoir data collected were its geographical and administrative locations. Intrinsic characteristics of the pigs collected were sex, physiological status, and body condition score [17].

### Data management and analysis

The data was organized using MS Excel. The apparent seroprevalence with a 95% confidence interval was calculated using Epitools (epitools.ausvet.com.au), considering an imperfect test (ELISA test diagnostic sensitivity of 99% and specificity of 99.5%). Descriptive analysis and graphical presentation were performed using GraphPad Prism (v. 9.5.1).

## Results

### Characteristics of sampled pigs

The 218 samples were finisher pigs with relatively equal sex distribution (female-to-male ratio of 1), and the majority (91.28%) had an ideal body condition score (Table 2). Samples were collected relatively evenly from the north (54.13%) and south (45.87%) parts of Leyte province. However, twice of the samples were from municipalities (66.97%) compared to cities (33.03%), reflecting the geographical characteristics of Leyte, a rural area where the population relies on farming and fishing.

### Farm-level and pig-level seroprevalence

**Figure 2.**
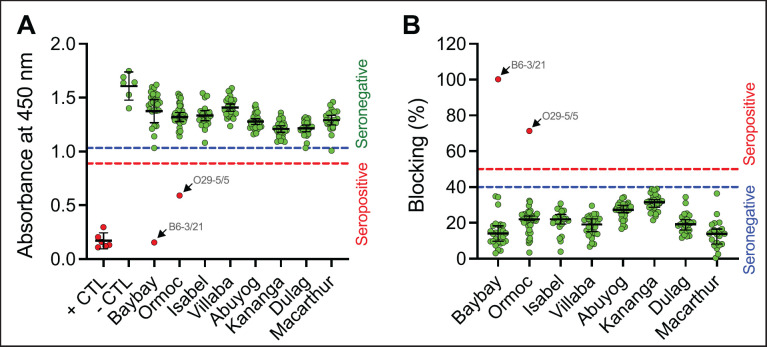
Blocking ELISA results in detecting antibodies of VP72 ASF virus proteins. (A) Results based on absorbance value at 450 nm. (B) Results according to the calculated blocking percentage. Note. Error bar: mean with 95% CI; Dots: values for each sample and control (CTL); Interrupted lines represent the cutoff values; B6-3/21 is the 6th selected pig serum sample from Baybay City abattoir collected on March 21, 2023; O29-5/5 is the 29th pig serum sample from Ormoc City abattoir collected on May 5, 2023.

Between March and June 2023, ASF (VP72) antibodies were detected in pigs for slaughter from abattoirs in Baybay and Ormoc cities (Fig. 2), showing a seroprevalence of 3.57% (1/28 pigs) and 2.27% (1/44 pigs), respectively. The seropositive pigs were all females with ideal body condition scores. An apparent absence of ASF antibodies was observed among pigs for slaughter in Isabel, Villaba, Abuyog, Kananga, Dulag, and Macarthur. At the farm level, the seroprevalence was 2.56% (95% CI: 0.71%–8.88%), while the individual-level seroprevalence was 0.91% (95% CI: 0.25%–3.27%) (Table 3).

## Discussion

The non-reporting of ASF outbreaks and the selling of pigs from outbreak zones have been implicated in the spread of ASF infection. With these possible scenarios occurring in Philippine farm settings [10], we hypothesize that recovered pigs from ASF or those exposed to ASF infection are brought to the abattoir for slaughter and human consumption. To address our hypothesis, we tested pig serum samples using a blocking ELISA method to assess the prevalence of antibodies against ASFV (VP72) in apparently healthy pigs for slaughter. This method uses a commercial ELISA kit (INGEZIM PPA COMPAC 11.PPA. K3; Ingenasa, Spain) approved for use by the European Union Reference Laboratory for ASF (EURL-ASF) and recommended by the World Organization for Animal Health [18].

**Table 1.** Farms and pig samples analyzed for ASF seroprevalence in Leyte, Philippines.

Municipalities or cities	Number of farm	Number of pig sample
Northern part		
Isabel	16	21
Kananga	1	25
Ormoc	15	44
Villaba	4	28
Southern part		
Abuyog	8	28
Baybay	19	28
Dulag	7	23
Macarthur	8	21
Total	78	218

**Table 2.** Descriptive analysis of pigs for slaughter tested for ASF antibody (n = 218).

Variables	*n*	%	ASF+	ASF-
Geographical location				
Northern part	118	54.13	1	117
Southern part	100	45.87	1	99
Administrative location				
City	72	33.03	2	70
Municipality	146	66.97	0	146
Pig Sex				
Female	111	50.92	2	109
Male	107	49.08	0	107
Body condition score				
Emaciated	0	0	0	0
Thin	5	2.29	0	5
Ideal	199	91.28	2	197
Fat	14	6.42	0	14
Overly fat	0	0	0	0

This study demonstrated the presence of ASF antibodies in pigs for slaughter during a non-outbreak period in Leyte, Philippines, which is the first research-based report in the study sites independent from the surveillance conducted by the Local Government Unit (LGU) and Department of Agriculture—Bureau of Animal Industry (DA-BAI). Here, the pig-level seroprevalence in eight selected abattoirs was around 1%, while the farm-level seroprevalence was about 3%. Albeit at low levels, the detection of ASF antibodies among slaughtered pigs implies that pigs are exposed to the virus from the farm of origin, indicating that ASF remains unreported or undiagnosed on some farms. One possible reason for low seroprevalence is the months of sample collections, which correspond to the hot dry season (March and May). Spatiotemporal patterns of ASF in the Philippines based on 2019–2022 data revealed that ASF outbreaks are lowest during the hot and dry months, with possible explanations on virus sensitivity to temperature and infrequent pig movements between farms and administrative borders [21]. Assuming the presence of unreported outbreaks in the study sites, the low seroprevalence could also be explained by the high mortality rates caused by the pathogenic ASFV (genotype II) present in the Philippines [19,20], meaning those remaining pigs that survived from ASF or were exposed to the virus were likely sold or delivered to the abattoir. The level of ASF seropositivity among pigs for slaughter in this study is comparable to the neighboring Southeast Asian country of Cambodia, showing a low ASF seroprevalence (2.6%) in pigs for slaughter in Phnom Penh. By contrast, ASF seroprevalence in some countries is high in apparently healthy pigs and pigs for slaughter, possibly indicating chronic infection associated with low pathogenic genotypes (e.g., ASFV genotypes I and IX) [22,23], which have not been reported in the Philippines.

This study found seropositive cases of pigs for slaughter delivered to city abattoirs, while pigs examined in municipal abattoirs showed seronegative results. City abattoirs offer services to farm owners or middlemen whose animals are outsourced from other locations. By contrast, pigs slaughtered in municipal abattoirs typically come from local pig producers whose sources and markets are limited within the municipality. A previous report has shown that, in the past ten years, significant numbers of live pigs in Leyte were derived from various provinces in Mindanao, the southern part of the Philippines [24]. Of note, several municipalities in Mindanao and Visayas remained ASF-positive from the time of sample collection and as of the writing [5]. However, there were no reports of active ASF cases on Leyte Island between March and June 2023 (the sample collection time). In fact, active cases were concentrated in neighboring provinces in the Visayas, namely Cebu and Northern Samar, and in Mindanao, such as Surigao del Norte and Surigao del Sur [25]. In essence, seropositive pigs in this study were detected in administrative areas with no official outbreak reports from DA-BAI. This finding may indicate a potential loophole in the implementation of the existing ASF prevention and control policy, as seropositive pigs appear to be transported from outbreak zones to non-outbreak zones. However, we cannot rule out the possibility of unreported sporadic cases in supposed areas without active ASF cases, implying a lack of active surveillance among LGUs. It is worth noting that ASF confirmatory tests (e.g., real-time polymerase chain reaction) performed by DA-BAI accredited laboratories are needed to officially declare an ASF outbreak according to government policy [26].

**Table 3.** Seroprevalence of ASF in cities and municipalities (78 farms and 218 pigs) in Leyte Island.

City or municipality	Farms	Seropositive samples	Pig samples	Apparent prevalence (%)	95% CI
Baybay	19	1	28	3.57	0.63–17.71
Ormoc	15	1	44	2.27	0.40–11.81
Isabel	16	0	21	0.00	0.00–15.46
Villaba	4	0	28	0.00	0.00–12.06
Abuyog	8	0	28	0.00	0.00–12.06
Kananga	1	0	25	0.00	0.00–13.32
Dulag	7	0	23	0.00	0.00–14.31
Macarthur	8	0	21	0.00	0.00–15.46
Overall data					
Farm level	78	2	-	2.56	0.71–8.88
Animal level	-	2	218	0.92	0.25–3.28

The apparent prevalence with a 95% Confidence Interval (Wilson CI) was calculated using epitools.ausvet.com.au, given the ELISA test sensitivity of 99% and specificity of 99.5%.

Overall, our results imply that, in selected abattoirs in Leyte, a few pigs brought for slaughter were exposed to ASF infection at the farm of origin. This means that ASFV may still be circulating in areas declared to have no active cases, suggesting potential loopholes in disease recognition and case reporting at the farm level and the possible lack of active surveillance at the LGU and DA-BAI levels. It is also worth investigating whether ASF is becoming endemic on the island. To circumvent the impact of ASF, future work should focus on co-creating strategies among stakeholders to mitigate its spread on Leyte Island. These include mechanisms to encourage early case reporting and the proper implementation of biosecurity measures.

## Conclusion

This study demonstrates the presence of ASF antibodies at low seroprevalence among pigs for slaughter during a non-outbreak period in Leyte, Philippines. The detection of ASF antibodies indicates the exposure of pigs to the virus from the farm of origin, implying that ASF remains unreported or undiagnosed on some farms. Thus, there is a need to improve the government’s surveillance system on ASF. It is also recommended that stakeholders be capacitated and positively engaged in disease recognition and early case reporting to control the spread of ASF in the country.

## List of abbreviations

ASF, African swine fever; ASFV, African swine fever virus; CVM, College of Veterinary Medicine; DA-BAI, Department of Agriculture-Bureau of Animal Industry; ELISA, Enzyme-linked immunosorbent assay; LGU, Local Government Unit.
